# Cu_9_S_5_/Gel-Derived TiO_2_ Composites for Efficient CO_2_ Adsorption and Conversion

**DOI:** 10.3390/gels11090711

**Published:** 2025-09-04

**Authors:** Shuai Liu, Yang Meng, Zhengfei Chen, Jiefeng Yan, Fuyan Gao, Tao Wu, Guangsuo Yu

**Affiliations:** 1 Institute of Clean Coal Technology, East China University of Science and Technology, Shanghai 200237, China; shuai.liu@nbt.edu.cn; 2 School of Mechatronics and Energy Engineering, NingboTech University, Ningbo 315100, China; zhengfei.chen@nbt.edu.cn; 3 Ningbo Key Laboratory of Urban Environmental Pollution Control, CAS Haixi Industrial Technology Innovation Center in Beilun, Ningbo 315830, China; ymeng@iue.ac.cn; 4 College of Science & Technology, Ningbo University, Ningbo 315100, China; yanjiefeng@nbu.edu.cn; 5 Nottingham Ningbo China Beacons of Excellence Research and Innovation Institute, 211 Xingguang Road, Ningbo 315048, China; tao.wu@nottingham.edu.cn

**Keywords:** Cu_9_S_5_/TiO_2_ gel composites, CO_2_ adsorption and conversion, methane selectivity, density functional theory (DFT)

## Abstract

Engineering phase-selective gel composites presents a promising route to enhance both CO_2_ adsorption and conversion efficiency in photocatalytic systems. In this work, Cu_9_S_5_/TiO_2_ gel composites were synthesized via a hydrazine-hydrate-assisted hydrothermal method, using TiO_2_ derived from a microwave-assisted sol–gel process. The resulting materials exhibit a porous gel-derived morphology with highly dispersed Cu_9_S_5_ nanocrystals, as confirmed by XRD, TEM, and XPS analyses. These structural features promote abundant surface-active sites and interfacial contact, enabling efficient CO_2_ adsorption. Among all samples, the optimized 0.36Cu_9_S_5_/TiO_2_ composite achieved a methane production rate of 34 μmol·g^−1^·h^−1^, with 64.76% CH_4_ selectivity and 88.02% electron-based selectivity, significantly outperforming Cu_9_S_8_/TiO_2_ synthesized without hydrazine hydrate. This enhancement is attributed to the dual role of hydrazine: facilitating phase transformation from Cu_9_S_8_ to Cu_9_S_5_ and modulating the interfacial electronic environment to favor CO_2_ capture and activation. DFT calculations reveal that Cu_9_S_5_/TiO_2_ effectively lowers the energy barriers of critical intermediates (*COOH, *CO, and *CHO), enhancing both CO_2_ adsorption strength and subsequent conversion to methane. This work demonstrates a gel-derived composite strategy that couples efficient CO_2_ adsorption with selective photocatalytic reduction, offering new design principles for adsorption–conversion hybrid materials.

## 1. Introduction

The rising concentration of atmospheric carbon dioxide (CO_2_), driven by intensified fossil fuel consumption and anthropogenic emissions, poses a critical threat to global climate stability. As the primary greenhouse gas, CO_2_ contributes significantly to global warming, environmental degradation, and energy imbalance [1,2,3]. In light of these challenges, developing sustainable and efficient CO_2_ conversion strategies has become an urgent imperative for achieving carbon neutrality and promoting renewable energy technologies [4,5].

Among various emerging technologies, photocatalytic CO_2_ reduction (CO_2_RR) offers a green, solar-driven route to convert CO_2_ into value-added fuels such as methane (CH_4_), ethylene (C_2_H_4_), methanol (CH_3_OH), and carbon monoxide (CO) [5,6,7,8,9,10] This artificial photosynthetic process utilizes semiconductor photocatalysts to activate and reduce CO_2_ under light irradiation, thereby enabling both carbon recycling and solar-to-chemical energy conversion. However, the practical application of photocatalytic CO_2_RR remains hindered by several intrinsic limitations, particularly those related to low quantum efficiency, poor CO_2_ adsorption, and product selectivity [11,12].

Titanium dioxide (TiO_2_), a widely studied photocatalyst, suffers from a wide bandgap (~3.3 eV) that restricts its optical response to the ultraviolet spectrum, as well as the rapid recombination of photogenerated charge carriers [13,14]. To overcome these drawbacks, composite photocatalysts have been extensively explored. By coupling TiO_2_ with visible-light-active materials such as metal oxides, sulfides, or nitrides, the resulting heterostructures exhibit improved light harvesting, interfacial charge transfer, and redox kinetics [15,16].

Copper-based sulfides (e.g., CuS, Cu_9_S_8_, Cu_9_S_5_, Cu_2_S) have attracted particular attention due to their narrow bandgaps, strong CO_2_ adsorption affinity, and suitable conduction band positions for CO_2_RR [17,18,19,20]. Among them, Cu_9_S_5_ exhibits enhanced visible light absorption and possesses favorable catalytic sites for deep reduction pathways toward methane [21,22,23]. Controlling the phase, dispersion, and interfacial interaction between Cu_9_S_5_ and the support remains essential for optimizing CO_2_ adsorption and selective CH_4_ formation [24,25,26]. However, despite extensive studies on Cu-based sulfides, no prior report has systematically combined phase-selective modulation (Cu_9_S_8_ → Cu_9_S_5_) with a gel-derived TiO_2_ matrix to simultaneously enhance CO_2_ adsorption and CH_4_ selectivity.

Hydrazine hydrate (N_2_H_4_·H_2_O), a strong reducing agent, offers unique advantages in tailoring the phase composition and surface electronic states of metal sulfide catalysts. When used during hydrothermal synthesis, the accurate addition of hydrazine can promote a desired phase transition and modulate the catalyst–support interface [27,28,29,30]. For example, Yang et al. demonstrated that hydrazine can effectively regulate defect states and promote charge separation in composite photocatalysts for hydrogen evolution, highlighting its potential in photocatalytic systems. Similarly, Sattigeri et al. reported that a hydrazine–sulfur complex can stabilize ZnS quantum dots and enhance their photocatalytic activity, underlining the role of hydrazine in controlling phase purity and nanostructure. In addition, Li et al. showed that hydrazine hydrate reduction could induce oxygen vacancies in Co_3_O_4_ nanosheets, thereby tuning the electronic structure and improving catalytic properties. Zhang et al. further confirmed that hydrazine-induced oxygen vacancies in MoO_3_ nanobelts significantly accelerated charge transfer and boosted electrochemical kinetics, offering a broader insight into the versatility of hydrazine in phase and defect engineering. Inspired by these findings, hydrazine treatment is expected to have great potential in facilitating the phase transition from Cu_9_S_8_ to Cu_9_S_5_, thereby enabling precise phase control and interfacial optimization in Cu-based sulfide photocatalysts. Furthermore, using gel-derived TiO_2_ prepared via a microwave-assisted sol–gel route provides a porous and high-surface-area matrix, ideal for CO_2_ adsorption and intimate interfacial contact with Cu_9_S_5_ domains [31].

Subsequently, in this study, we present a novel strategy for synthesizing Cu_9_S_5_/TiO_2_ composites by employing a hydrazine-hydrate-assisted hydrothermal method. This approach not only induces the phase transition from Cu_9_S_8_ to Cu_9_S_5_ but also enhances the dispersion and crystallinity of Cu_9_S_5_ on TiO_2_. Additionally, we introduce a gel-derived composite framework for TiO_2_, prepared via a microwave-assisted sol-gel method, which improves both CO_2_ adsorption and the interfacial contact between Cu_9_S_5_ and TiO_2_. This strategy integrates the advantages of both CO_2_ adsorption and selective conversion functionalities into a single composite material, addressing two critical challenges in photocatalytic CO_2_ reduction. Furthermore, density functional theory (DFT) calculations are employed to investigate the electronic structure and reaction pathways of Cu_9_S_5_/TiO_2_ composites, offering insights into the enhanced methane selectivity observed in these materials. The combination of hydrazine-induced phase transformation and gel-based composite design provides a highly efficient, selective, and stable photocatalyst for CO_2_ reduction.

## 2. Results and Discussion

### 2.1. Structural Features of Gel-Derived Cu_9_S_5_/TiO_2_ Composites

**Figure 1:**
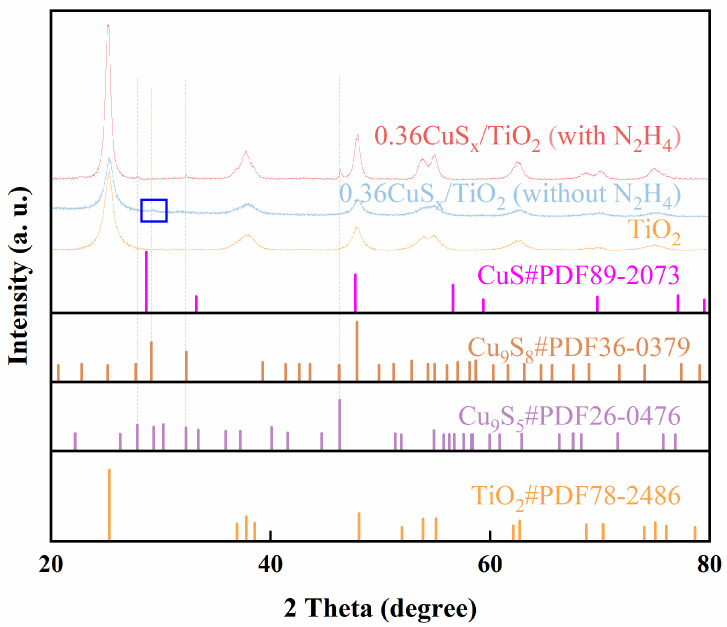
X-ray diffraction (XRD) patterns of the gel-derived TiO_2_, 0.36CuS_x_/TiO_2_ (with N_2_H_4_), and 0.36CuS_x_/TiO_2_ (without N_2_H_4_).

The phase composition and crystallinity of the gel-derived CuS_x_/TiO_2_ composites, both with and without the inclusion of hydrazine hydrate (N_2_H_4_), were thoroughly examined using X-ray diffraction (XRD), as illustrated in Figure 1. The XRD pattern of pure TiO_2_ exhibits characteristic peaks at 25.3°, 37.8°, 48.1°, and 55.1°, corresponding to the anatase phase (PDF#78-2486). The XRD peaks of TiO_2_ in the 0.36CuS_x_/TiO_2_ sample synthesized with hydrazine hydrate are sharper and more defined, indicating an enhancement in the crystallinity of TiO_2_. This improvement in TiO_2_ crystallinity is likely due to the better phase formation and reduced defects induced by the presence of hydrazine hydrate, which helps to stabilize the TiO_2_ structure during the hydrothermal synthesis.

For the 0.36CuS_x_/TiO_2_ sample synthesized without hydrazine hydrate, additional diffraction peaks appear at 29.15°, which correspond to the Cu_9_S_8_ phase (PDF#36-0379). The main peak at 47.9° overlaps with those of TiO_2_, suggesting that the TiO_2_ structure remains largely intact while Cu_9_S_8_ is formed. This confirms that the Cu_9_S_8_ phase is the dominant copper sulfide species when hydrazine hydrate is not used in the synthesis. In contrast, the 0.36CuS_x_/TiO_2_ sample synthesized with hydrazine hydrate shows distinct peaks at 27.9°, 32.4°, and 46.4°, which can be indexed to Cu_9_S_5_ (PDF#26-0476). These peaks do not overlap with TiO_2_, indicating the formation of Cu_9_S_5_ and confirming that hydrazine hydrate influences the phase transition from Cu_9_S_8_ to Cu_9_S_5_. Importantly, no CuS species were observed in any of the samples, highlighting that the formation of Cu_9_S_8_ or Cu_9_S_5_ is strictly controlled by the presence of hydrazine hydrate under the hydrothermal conditions. These results emphasize the critical role of hydrazine hydrate in modulating the copper sulfide phase in CuS_x_/TiO_2_ composites. In the next sections, the samples synthesized with hydrazine hydrate are referred to as Cu_9_S_5_/TiO_2_, while those synthesized without hydrazine hydrate are referred to as Cu_9_S_8_/TiO_2_.

**Figure 2:**
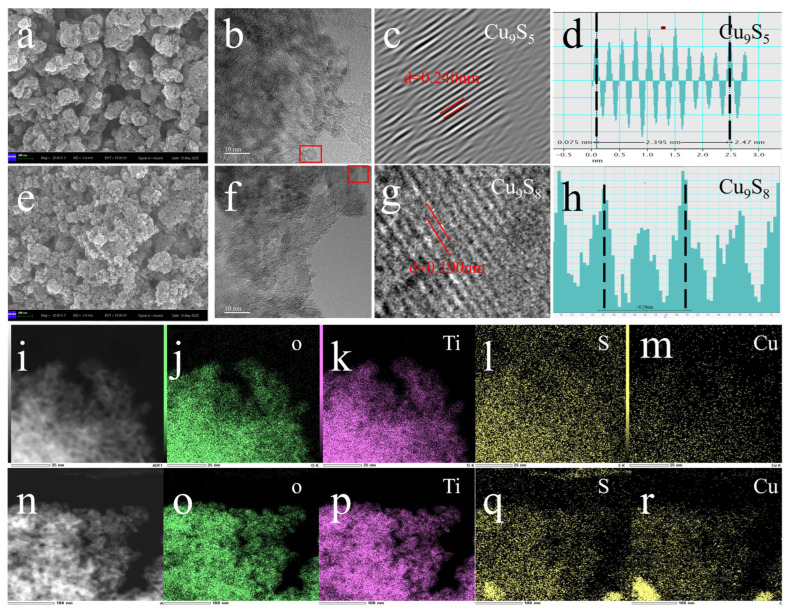
Morphology and elemental distribution analysis of the gel-derived Cu_9_S_5_/TiO_2_ and Cu_9_S_8_/TiO_2_ composites. (**a**) SEM image of Cu_9_S_5_/TiO_2_ composite, (**b**) TEM image of Cu_9_S_5_/TiO_2_ composite, (**c**) high-resolution TEM image of Cu_9_S_5_, (**d**) fast Fourier transform (FFT) pattern of Cu_9_S_5_, (**e**) SEM image of Cu_9_S_8_/TiO_2_ composite, (**f**) TEM image of Cu_9_S_8_/TiO_2_, (**g**) High-resolution TEM image of Cu_9_S_8_, (**h**) FFT pattern of Cu_9_S_8_, (**i**) TEM image of Cu_9_S_5_/TiO_2_ composite (overview). (**j**–**m**) EDS mapping of O, Ti, S, Cu in Cu_9_S_5_/TiO_2_ composite, (**n**) TEM image of Cu_9_S_8_/TiO_2_ composite (overview), and (**o**–**r**) EDS mapping of O, Ti, S, Cu in Cu_9_S_8_/TiO_2_ composite.

The morphology and microstructure of the gel-derived Cu_9_S_5_/TiO_2_ and Cu_9_S_8_/TiO_2_ composites were investigated using a combination of SEM, TEM, high-resolution TEM (HRTEM), and energy-dispersive X-ray spectroscopy (EDS) mapping, as illustrated in Figure 2. For the Cu_9_S_5_/TiO_2_ composite synthesized with hydrazine hydrate, the SEM image (Figure 2a) reveals a relatively uniform distribution of Cu_9_S_5_ particles across the TiO_2_ surface. The TEM image (Figure 2b) further confirms that the Cu_9_S_5_ domains are evenly dispersed without significant agglomeration. High-resolution TEM analysis (Figure 2c) displays distinct lattice fringes with an interplanar spacing of approximately 0.240 nm, which can be indexed to the (101) plane of Cu_9_S_5_, indicating the formation of a well-crystallized phase. The corresponding FFT pattern (Figure 2d) shows sharp diffraction spots, consistent with a highly ordered crystalline structure. A low-magnification TEM overview (Figure 2i) supports the homogeneous distribution of Cu_9_S_5_ on the TiO_2_ support. In contrast, the Cu_9_S_8_/TiO_2_ composite prepared without hydrazine hydrate exhibits less favorable morphology. As shown in the SEM image (Figure 2e), Cu_9_S_8_ particles are more aggregated and less uniformly spread across the TiO_2_ surface. TEM observation (Figure 2f) also reveals uneven distribution and particle clustering. The HRTEM image (Figure 2g) shows lattice fringes with a spacing of 0.190 nm, corresponding to the (111) plane of Cu_9_S_8_, confirming the formation of the Cu_9_S_8_ phase. However, the associated FFT pattern (Figure 2h) is less distinct, suggesting relatively lower crystallinity compared to the Cu_9_S_5_ counterpart. The overall particle arrangement in the overview TEM image (Figure 2n) further confirms the irregular distribution of Cu_9_S_8_ domains.

Elemental mapping via EDS was employed to examine the spatial distribution of key elements in both composites. For Cu_9_S_5_/TiO_2_, the EDS maps (Figure 2j–m) clearly show the co-localization of Ti, Cu, and S, with Cu and S signals uniformly distributed across the TiO_2_ substrate, consistent with the homogeneous dispersion of Cu_9_S_5_. The oxygen map further supports the presence of TiO_2_ throughout the sample. In comparison, the EDS maps of Cu_9_S_8_/TiO_2_ (Figure 2o–r) show more localized and concentrated Cu and S signals, corresponding to the Cu_9_S_8_ aggregates, while Ti and O remain uniformly distributed.

Collectively, the X-ray diffraction and transmission electron microscopy analyses confirm the formation of Cu_9_S_5_ in the composites. The role of hydrazine hydrate in promoting the phase transition from Cu_9_S_8_ to Cu_9_S_5_ can be summarized in the following reaction scheme. In the first stage, Cu_9_S_8_ is initially formed during the synthesis process in the absence of hydrazine hydrate. In the second stage, hydrazine hydrate acts as a strong reducing agent, donating electrons to Cu_9_S_8_ and reducing Cu^2+^ to Cu^+^, which then combine with sulfur to form Cu_9_S_5_. The Cu_9_S_5_/TiO_2_ composite exhibits superior microstructural uniformity and phase clarity, which are expected to enhance interfacial contact and charge separation during photocatalysis, aligning with the enhanced performance discussed in later sections.

**Figure 3:**
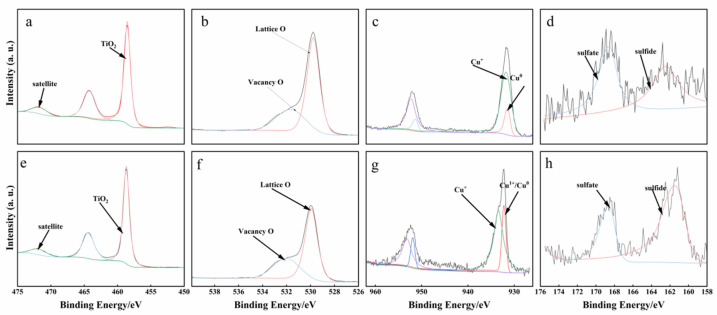
High-resolution XPS spectra of the gel-derived Cu_9_S_5_/TiO_2_ composites and Cu_9_S_8_/TiO_2_: (**a**,**e**) Ti 2p, (**b**,**f**) O 1s, (**c**,**g**) Zn 2p, and (**d**,**h**) S 2p.

To gain deeper insight into the surface composition and electronic states of the gel-derived Cu_9_S_5_/TiO_2_ and Cu_9_S_8_/TiO_2_ composites, X-ray photoelectron spectroscopy (XPS) was performed on both materials as shown in Figure 3. The XPS spectra of Ti 2p, O 1s, Cu 2p, and S 2p regions were analyzed to investigate the chemical environment of titanium, oxygen, copper, and sulfur species, which are crucial for understanding the photocatalytic behavior of these composites. The Ti 2p spectra of both composites exhibited a characteristic binding energy of 458.6 eV, which is typical for TiO_2_. This confirms that the TiO_2_ phase is preserved in both the Cu_9_S_5_/TiO_2_ and Cu_9_S_8_/TiO_2_ composites. The spectra also show a satellite peak at lower binding energies, which is a signature feature of TiO_2_, indicating that the introduction of copper sulfides did not significantly alter the TiO_2_ structure. There were no significant shifts in the Ti 2p binding energy, further suggesting the stability of the TiO_2_ phase. In the O 1s region, two distinct peaks were observed corresponding to lattice oxygen (Lattice O) and vacancy oxygen (Vacancy O), with binding energies around 529 eV and 532 eV, respectively. These peaks are indicative of the oxygen species present in the TiO_2_ lattice and the oxygen vacancies that are often present in metal oxides. Both Cu_9_S_5_/TiO_2_ and Cu_9_S_8_/TiO_2_ show similar peak positions and relative intensities, suggesting that the incorporation of Cu_9_S_5_ or Cu_9_S_8_ did not cause significant changes in the oxygen species or the oxygen vacancies within the TiO_2_ lattice.

The Cu 2p spectra showed notable differences between the two composites. For Cu_9_S_5_/TiO_2_, the Cu 2p spectrum exhibited peaks corresponding to Cu^0^ at 932.4 eV and Cu^+^ at 932.7 eV, indicating the presence of both Cu(I) and Cu(0) species. This suggests that hydrazine hydrate, used during synthesis, played a key role in reducing Cu^2+^ to Cu^0^ and Cu^+^, both of which are important for enhancing photocatalytic activity. In contrast, the Cu_9_S_8_/TiO_2_ composite exhibited peaks corresponding to Cu^+^ at 932.73 eV and Cu^2+^ at 933.6 eV, indicating a higher proportion of Cu^2+^ species. This suggests that in the absence of hydrazine hydrate, Cu^2+^ remains more prevalent, which likely limits the photocatalytic activity compared to Cu_9_S_5_/TiO_2_. This further emphasizes the importance of hydrazine hydrate in reducing copper species and enhancing catalytic efficiency.

In the S 2p region, both Cu_9_S_5_/TiO_2_ and Cu_9_S_8_/TiO_2_ composites exhibited peaks corresponding to sulfate and sulfide species. The presence of sulfate at 168.6 eV suggests that sulfur is bonded to oxygen, possibly forming sulfate species due to surface oxidation or interaction with surface oxygen atoms from TiO_2_. The more intense sulfide peak at 161.4 eV indicates that sulfur primarily exists in its reduced state as sulfide. The higher intensity of the sulfide peak in both composites suggests that the sulfide species are more abundant and better dispersed on the surface. This finding is consistent with the energy dispersive spectroscopy (EDS) results, which showed a more homogeneous distribution of Cu_9_S_5_.

### 2.2. Optical and Photoelectrochemical Properties of Gel-Derived Cu_9_S_5_/TiO_2_ and Cu_9_S_8_/TiO_2_

**Figure 4:**
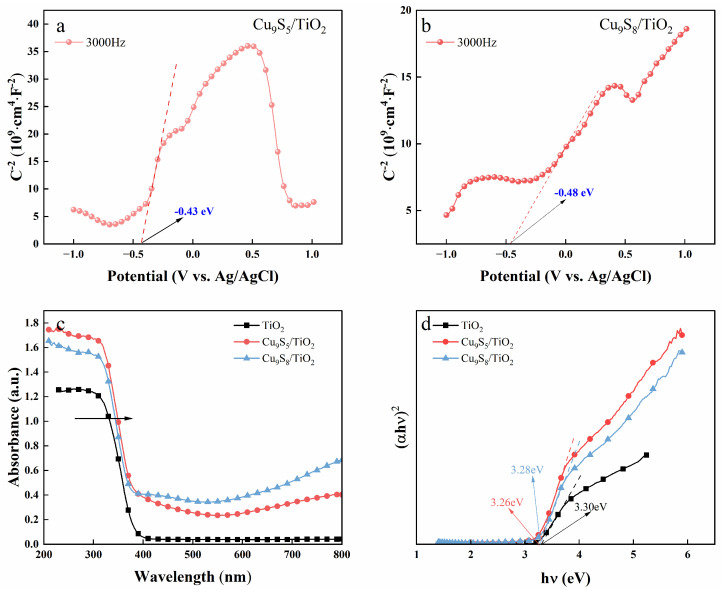
Optical properties of the gel-derived Cu_9_S_5_/TiO_2_ and Cu_9_S_8_/TiO_2_ composites. (**a**,**b**) Mott–Schottky plots of Cu_9_S_5_/TiO_2_ and Cu_9_S_8_/TiO_2_; (**c**,**d**) UV–vis absorption spectra and Tauc plots of Cu_9_S_5_/TiO_2_ and Cu_9_S_8_/TiO_2_ composites.

Figure 4 presents the optical properties of the gel-derived Cu_9_S_5_/TiO_2_ and Cu_9_S_8_/TiO_2_ composites, shedding light on their potential for photocatalytic applications. Figure 4a,b display the Mott–Schottky plots for Cu_9_S_5_/TiO_2_ and Cu_9_S_8_/TiO_2_ composites, respectively, used to determine the flat-band potential (E_fb) and semiconductor type. The plot for Cu_9_S_5_/TiO_2_ (Figure 4a) reveals a flat-band potential around −0.43 eV, indicative of n-type semiconductor characteristics, which is important for efficient electron transfer during photocatalytic reactions. Similarly, the Mott–Schottky plot for Cu_9_S_8_/TiO_2_ (Figure 4b) shows a flat-band potential of approximately −0.48 eV, confirming the n-type nature of this composite as well.

In Figure 4c,d, the optical properties of the composites are examined through UV–vis absorption spectra and Tauc plots. Figure 4c shows that TiO_2_ has a sharp absorption edge around 380 nm, typical of its wide band gap, which limits its absorption to the UV region. Both Cu_9_S_5_/TiO_2_ and Cu_9_S_8_/TiO_2_ composites exhibit enhanced absorption in the visible light range compared to pure TiO_2_. Cu_9_S_5_/TiO_2_ demonstrates a more prominent absorption in the visible region, extending well beyond 600 nm and up to 800 nm, indicating significantly improved visible light absorption. On the other hand, Cu_9_S_8_/TiO_2_ also absorbs in the visible region, but the absorption intensity is slightly lower than that of Cu_9_S_5_/TiO_2_, which suggests that Cu_9_S_5_/TiO_2_ has a better capacity for visible light utilization.

Figure 4d presents the Tauc plots, where the optical band gaps (Eg) of the composites are determined by plotting (αhν)^2^ versus hν. The optical band gap of Cu_9_S_8_/TiO_2_ is measured to be 3.28 eV, which is slightly smaller than that of TiO_2_ (3.30 eV), indicating enhanced visible light absorption due to the Cu_9_S_8_ phase. In contrast, the optical band gap of Cu_9_S_5_/TiO_2_ is even smaller, measured at 3.26 eV, confirming that Cu_9_S_5_ further reduces the band gap and enhances the ability of the composite to absorb visible light more efficiently.

Therefore, the results from the Mott–Schottky analysis and UV–vis absorption spectra show that the incorporation of Cu_9_S_5_ and Cu_9_S_8_ into TiO_2_ enhances their photocatalytic performance by improving visible light absorption. Cu_9_S_5_/TiO_2_ (3.26 eV) exhibits a smaller band gap than Cu_9_S_8_/TiO_2_ (3.28 eV), which enables better visible light absorption and higher photocatalytic efficiency, making both composites promising candidates for visible-light-driven photocatalysis.

**Figure 5:**
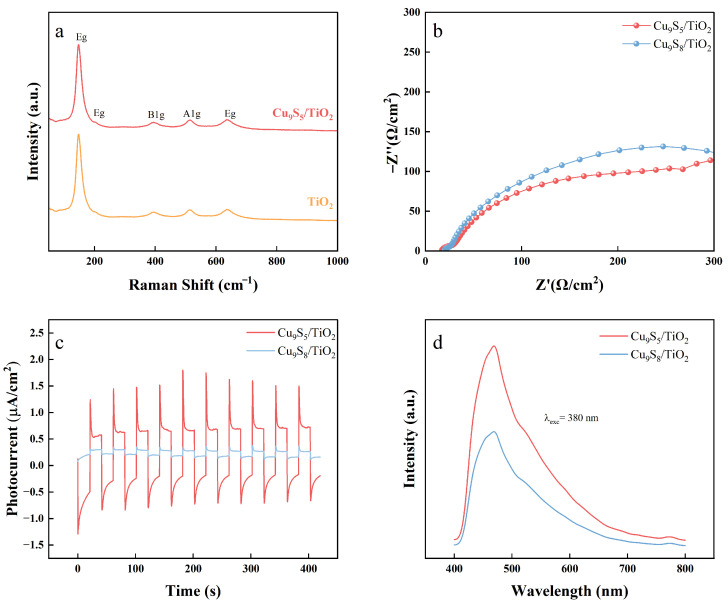
Structural and electrochemical properties of the gel-derived Cu_9_S_5_/TiO_2_ and Cu_9_S_8_/TiO_2_ Composites. (**a**) Raman spectra of Cu_9_S_5_/TiO_2_ and TiO_2_, (**b**) electrochemical impedance spectroscopy (EIS) Nyquist plots of Cu_9_S_5_/TiO_2_ and Cu_9_S_8_/TiO_2_ composites, (**c**) photocurrent responses of Cu_9_S_5_/TiO_2_ and Cu_9_S_8_/TiO_2_ under periodic light illumination, and (**d**) photoluminescence (PL) spectra of Cu_9_S_5_/TiO_2_ and Cu_9_S_8_/TiO_2_.

Figure 5 demonstrates the structural and electrochemical properties of TiO_2_ and Cu_9_S_5_/TiO_2_ composites, providing insights into their photocatalytic performance. As shown in Figure 5a, the Raman spectrum of TiO_2_ shows the characteristic peaks around 143 cm^−1^ and 445 cm^−1^, which correspond to the Eg and A1g vibrational modes of the TiO_2_ anatase phase. In contrast, the Cu_9_S_5_ spectrum exhibits an additional peak at approximately 200 cm^−1^, corresponding to the Cu-S vibrational mode, which confirms the successful incorporation of Cu_9_S_5_ into the composite.

In Figure 5b, the electrochemical impedance spectroscopy (EIS) results are shown for Cu_9_S_5_/TiO_2_ and Cu_9_S_8_/TiO_2_ composites. The Nyquist plots display the charge transfer resistance (Rct) of both composites. The Cu_9_S_5_/TiO_2_ composite shows a significantly lower Rct compared to Cu_9_S_8_/TiO_2_, suggesting enhanced charge carrier mobility and more efficient charge transfer in Cu_9_S_5_/TiO_2_. This lower impedance indicates better photocatalytic performance, as reduced resistance facilitates more effective electron transport during the photocatalytic process.

Figure 5c presents the photocurrent responses of Cu_9_S_5_/TiO_2_ and Cu_9_S_8_/TiO_2_ under periodic light illumination. The Cu_9_S_5_/TiO_2_ composite shows a stronger and more stable photocurrent response compared to Cu_9_S_8_/TiO_2_, which demonstrates the superior photoelectrochemical performance of Cu_9_S_5_/TiO_2_. This is due to the enhanced light absorption and efficient charge separation promoted by Cu_9_S_5_, which contributes to higher photocurrent generation under visible light irradiation.

Figure 5d shows the photoluminescence (PL) spectra of TiO_2_, Cu_9_S_5_/TiO_2_, and Cu_9_S_8_/TiO_2_ composites. The PL intensity of Cu_9_S_5_/TiO_2_ is significantly lower than that of TiO_2_ and Cu_9_S_8_/TiO_2_, indicating a reduced recombination of photogenerated electron–hole pairs. This reduced recombination enhances the photocatalytic efficiency of Cu_9_S_5_/TiO_2_, as more charge carriers are available for photocatalytic reactions.

Obviously, the incorporation of Cu_9_S_5_ improves charge transfer, reduces charge recombination, and enhances light absorption, making Cu_9_S_5_/TiO_2_ a promising composite for efficient photocatalytic applications.

### 2.3. Photocatalytic Performance and Selectivity Toward Methane

**Figure 6:**
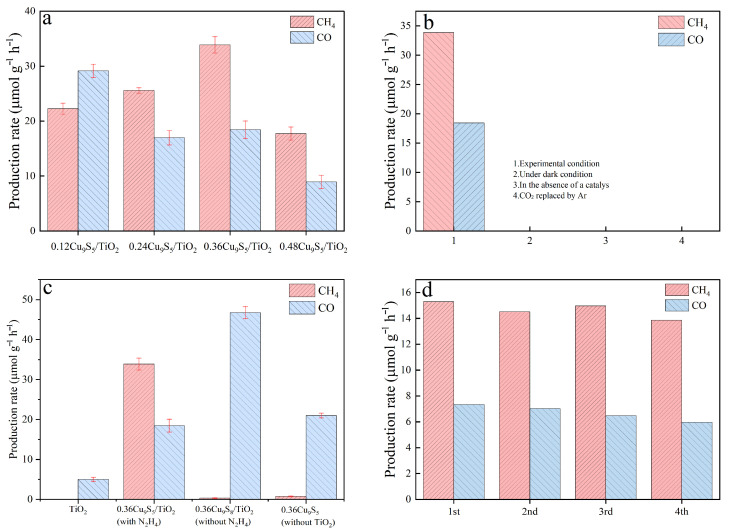
Performance analysis of the gel-derived Cu_9_S_5_/TiO_2_ composites for photocatalytic CO_2_ reduction. (**a**) The CO_2_ reduction performance of Cu_9_S_5_/TiO_2_ composites with varying Cu_9_S_5_ content (0.12Cu_9_S_5_/TiO_2_, 0.24Cu_9_S_5_/TiO_2_, 0.36Cu_9_S_5_/TiO_2_, and 0.48Cu_9_S_5_/TiO_2_) under standard experimental conditions. (**b**) Impact of different experimental conditions on the photocatalytic CO_2_ reduction of 0.36Cu_9_S_5_/TiO_2_ composite: (1) standard experimental conditions, (2) reaction under dark conditions, (3) in the absence of catalyst, and (4) CO_2_ replaced by Ar. (**c**) Photocatalytic CO_2_ reduction performance of TiO_2_, 0.36Cu_9_S_5_/TiO_2_ (with N_2_H_4_), 0.36Cu_9_S_8_/TiO_2_ (without N_2_H_4_), and 0.36Cu_9_S_5_/TiO_2_ (without TiO_2_). (**d**) Stability of the 0.36Cu_9_S_5_/TiO_2_ composite over four consecutive cycles of CO_2_ reduction.

Figure 6 presents a comprehensive analysis of the photocatalytic CO_2_ reduction performance of various Cu_9_S_5_/TiO_2_ composites under different experimental conditions, highlighting their efficiency and stability. Figure 6a shows the production rates of CH_4_ and CO from CO_2_ reduction by Cu_9_S_5_/TiO_2_ composites with varying Cu_9_S_5_ content (0.12Cu_9_S_5_/TiO_2_, 0.24Cu_9_S_5_/TiO_2_, 0.36Cu_9_S_5_/TiO_2_, and 0.48Cu_9_S_5_/TiO_2_). The results indicate that the 0.36Cu_9_S_5_/TiO_2_ composite exhibits the highest CH_4_ production rate, reaching approximately 34 μmol/g·h, followed by the 0.48Cu_9_S_5_/TiO_2_ composite, which produces 28 μmol/g·h of CH_4_. The 0.12Cu_9_S_5_/TiO_2_ and 0.24Cu_9_S_5_/TiO_2_ composites show lower CH_4_ production rates, around 20 μmol/g·h and 22 μmol/g·h, respectively. On the other hand, the CO production rates remain relatively consistent across all composites, with 0.36Cu_9_S_5_/TiO_2_ showing 15 μmol/g·h, the highest CO production rate among the composites. This suggests that a moderate amount of Cu_9_S_5_ (0.36 mmol) enhances the photocatalytic activity for CH_4_ production, while maintaining a relatively balanced CO generation.

Figure 6b compares the CO_2_ reduction performance of the 0.36Cu_9_S_5_/TiO_2_ composite under various experimental conditions. In Condition 1, under standard experimental conditions with light irradiation, the 0.36Cu_9_S_5_/TiO_2_ composite shows significant production of both CH_4_ (around 34 μmol·g^−1^·h^−1^) and CO (approximately 15 μmol·g^−1^·h^−1^). In Condition 2, when the reaction is conducted in the dark, the production of both CH_4_ and CO is almost negligible, demonstrating the essential role of light in driving the photocatalytic process. Condition 3, where no catalyst is used, results in zero CH_4_ and CO production, highlighting the need for an efficient catalyst to facilitate the CO_2_ reduction. Finally, in Condition 4, where CO_2_ is replaced by Ar, the reaction effectively ceases, further confirming that CO_2_ is crucial for the reaction to occur.

In Figure 6c, the photocatalytic CO_2_ reduction performance of various composites is shown: TiO_2_, 0.36Cu_9_S_5_/TiO_2_ with hydrazine hydrate (N_2_H_4_), 0.36Cu_9_S_8_/TiO_2_ without hydrazine hydrate, and 0.36Cu_9_S_5_/TiO_2_ without TiO_2_. The 0.36Cu_9_S_5_/TiO_2_ composite with hydrazine hydrate achieves the highest CH_4_ production rate of 34 μmol·g^−1^·h^−1^ and CO production rate of 15 μmol/g·h, significantly outperforming TiO_2_ (5 μmol·g^−1^·h^−1^ CO) and the 0.36Cu_9_S_8_/TiO_2_ without hydrazine hydrate (46 μmol·g^−1^·h^−1^ CO). The addition of hydrazine hydrate improves the separation of photogenerated charge carriers and increases the photocatalytic efficiency, leading to a higher CH_4_ production rate.

In addition, in order to evaluate the activity of our Cu_9_S_5_/TiO_2_ composites, we compared the results with representative copper sulfide–TiO_2_ systems reported in the literature. For example, a 0D/1D Cu_2-*x*_S/TiO_2_ S-scheme heterojunction exhibited a CH_4_ formation rate of 14.1 μmol·h^−1^, which was nearly 3.9 times higher than that of pristine TiO_2_ under similar conditions [32]. Another study on an in situ constructed Cu_2_S/TiO_2_ Schottky junction achieved CO and CH_4_ production rates of 6.71 μmol·g^−1^·h^−1^ and 1.20 μmol·g^−1^·h^−1^, corresponding to 1.41- and 5.71-fold enhancements relative to bare TiO_2_ [33]. For CuS/TiO_2_ composites, a photothermal–photocatalytic system utilizing 2 wt% CuS/TiO_2_ under full-spectrum irradiation demonstrated enhanced CO_2_ conversion efficiency due to the synergistic effect of CuS-induced photothermal heating [34]. To the best of our knowledge, no prior studies have directly investigated Cu_9_S_8_- or Cu_9_S_5_-based composites for CO_2_ adsorption or reduction. The only related work involving Cu_9_S_5_ is its combination with S,C,N-doped TiO_2_ for photocatalytic N_2_ fixation rather than CO_2_ conversion. Therefore, the present study provides a novel strategy by employing hydrazine-assisted phase transformation (Cu_9_S_8_ → Cu_9_S_5_) within a gel-derived TiO_2_ framework, enabling both enhanced CO_2_ adsorption and selective CH_4_ photoreduction.

Figure 6d demonstrates the stability of the 0.36Cu_9_S_5_/TiO_2_ composite in four consecutive cycles of CO_2_ reduction. The production of CH_4_ and CO remains relatively consistent across the cycles, with CH_4_ production at 34 μmol·g^−1^·h^−1^ and CO production at 15 μmol·g^−1^·h^−1^ in the first cycle, and only a slight decrease observed in subsequent cycles. This shows that the 0.36Cu_9_S_5_/TiO_2_ composite exhibits excellent stability and can be used for multiple cycles without significant degradation in photocatalytic performance. The minimal decrease in CH_4_ and CO production over the cycles suggests that the composite is highly durable, making it suitable for real-world applications in photocatalytic CO_2_ reduction.

In a word, Figure 6 demonstrates that the 0.36Cu_9_S_5_/TiO_2_ composite, especially with hydrazine hydrate treatment, is an efficient and stable photocatalyst for CO_2_ reduction, producing substantial amounts of CH_4_ and CO. The results also underscore the critical role of light, CO_2_, and catalyst in achieving efficient photocatalytic CO_2_ reduction. The excellent stability over four cycles further highlights the composite’s potential for practical applications in renewable energy and environmental sustainability.

**Figure 7:**
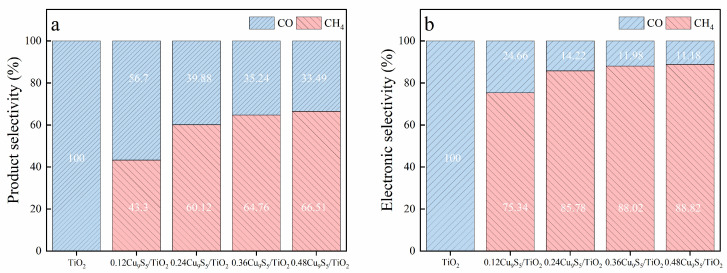
Selectivity analysis of CO_2_ photoreduction over the gel-derived TiO_2_ and Cu_9_S_5_/TiO_2_ composites. (**a**) Product selectivity for CH_4_ and CO; (**b**) calculated electronic selectivity based on electron consumption for respective products.

Figure 7a illustrates the product selectivity for CH_4_ and CO over the TiO_2_ and Cu_9_S_5_/TiO_2_ composites. For TiO_2_, no CH_4_ is produced (0%), and the product selectivity is entirely CO (100%). As the Cu_9_S_5_ content in the composites increases, the selectivity for CH_4_ significantly improves. Specifically, the 0.48Cu_9_S_5_/TiO_2_ composite shows the highest CH_4_ selectivity at 66.51%, accompanied by a CO selectivity of 33.49%. The 0.36Cu_9_S_5_/TiO_2_ composite also shows a notable CH_4_ selectivity of 64.76%, with CO selectivity at 35.24%, indicating that the addition of Cu_9_S_5_ enhances CH_4_ formation. The 0.24Cu_9_S_5_/TiO_2_ composite displays a balanced selectivity of 60.12% for CH_4_ and 39.88% for CO, while the 0.12Cu_9_S_5_/TiO_2_ composite exhibits 43.3% CH_4_ selectivity and 56.7% CO selectivity. These results demonstrate the progressive role of Cu_9_S_5_ in shifting the product distribution towards CH_4_.

Figure 7b presents the electronic selectivity based on electron consumption for CH_4_ and CO. The 0.36Cu_9_S_5_/TiO_2_ composite exhibits the highest electronic selectivity for CH_4_ at 88.02%, confirming its role in promoting efficient electron utilization for CH_4_ formation. The 0.48Cu_9_S_5_/TiO_2_ composite follows with an electronic selectivity for CH_4_ of 88.82% and 11.18% for CO. In contrast, TiO_2_ and the other Cu_9_S_5_/TiO_2_ composites demonstrate significantly higher electronic selectivity for CO, reflecting the greater electron demand for CO formation. Specifically, the 0.12Cu_9_S_5_/TiO_2_ composite shows an electronic selectivity for CH_4_ of 75.34%, and 0.24Cu_9_S_5_/TiO_2_ shows 85.77% for CH_4_, indicating an improvement in electron efficiency with increasing Cu_9_S_5_ content.

These findings highlight the significant effect of Cu_9_S_5_ content on both product selectivity and electronic efficiency in CO_2_ photoreduction. The 0.36Cu_9_S_5_/TiO_2_ composite achieves the best balance of CH_4_ and CO selectivity with 64.76% CH_4_ selectivity and 35.24% CO selectivity, while demonstrating the highest electronic selectivity for CH_4_ at 88.02%. This makes the 0.36Cu_9_S_5_/TiO_2_ composite a promising photocatalyst for selective CO_2_ reduction towards methane, offering an efficient electron utilization and favorable product distribution.

### 2.4. DFT Calculations and Heterojunction Analysis

**Figure 8:**
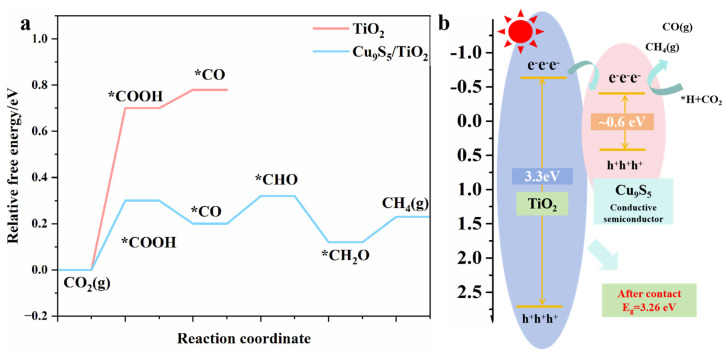
(**a**) DFT-calculated reaction free energy profiles for CO_2_ photoreduction over TiO_2_ and Cu_9_S_5_/TiO_2_ interfaces. (**b**) Band structure diagram of TiO_2_ and Cu_9_S_5_/TiO_2_ based on experimental flat-band potentials and Tauc-derived bandgaps. The asterisk * denotes the adsorbed state.

To clarify the origin of the enhanced CH_4_ selectivity in Cu_9_S_5_/TiO_2_ composites, we combined DFT calculations and band structure characterization to reveal how interfacial energetics and charge dynamics modulate CO_2_ reduction pathways. Figure 8 presents the DFT-calculated reaction free energy profiles and band structure diagrams for TiO_2_ and Cu_9_S_5_/TiO_2_ composites, offering a detailed insight into the photocatalytic CO_2_ reduction mechanism, particularly focusing on the intermediates COOH, CO, and CHO, which are critical to methane (CH_4_) formation.

Figure 8a illustrates the reaction free energy profiles for CO_2_ photoreduction over TiO_2_ and Cu_9_S_5_/TiO_2_ composites, highlighting the key intermediates that drive the CO_2_ reduction process. The energy profiles for COOH, CO, and CHO intermediates play a significant role in determining the overall efficiency of the reaction, particularly in methane generation. For TiO_2_ (red line), the energy barrier for the COOH intermediate is relatively high at 0.8 eV, indicating a less favorable formation of CO and CHO, which are essential intermediates for methane production. The CO intermediate has a high energy value of 0.78 eV, while the CHO intermediate is similarly high, further limiting the pathway toward efficient methane formation.

In contrast, the Cu_9_S_5_/TiO_2_ composite (blue line) significantly reduces the energy barriers for the critical intermediates. The COOH intermediate’s energy is lowered to 0.3 eV, facilitating easier conversion to CO and CHO. This reduced barrier encourages the formation of CO (with an energy value of 0.32 eV) and CHO (with an energy value of 0.12 eV), both of which are key precursors to methane formation. The CHO intermediate is particularly important, as its strong adsorption is a key step in methane production. Additionally, the strong adsorption of CO and CHO intermediates at the active sites on the Cu_9_S_5_/TiO_2_ composite provides an ideal pathway for efficient methane production. This is evident in the lower energy profile for CH_4_ formation on Cu_9_S_5_/TiO_2_ compared to TiO_2_. The lower energy for CH_4_ formation on Cu_9_S_5_/TiO_2_ directly supports its higher photocatalytic activity for CO_2_ reduction to methane.

Figure 8b shows the band structure of TiO_2_ and Cu_9_S_5_/TiO_2_ composites, further supporting the enhanced photocatalytic performance. TiO_2_ exhibits a conduction band edge at 0.54 eV, while the Cu_9_S_5_/TiO_2_ composite has a lower conduction band edge at 0.43 eV, which facilitates more efficient electron transfer. This lowering of the conduction band edge enhances electron mobility, promoting better separation of charge carriers and leading to more efficient reduction of CO_2_ to CH_4_.

Together, these results demonstrate that the Cu_9_S_5_/TiO_2_ composite not only facilitates the efficient formation of key intermediates such as CO and CHO but also significantly lowers the energy barriers for CH_4_ production. This makes Cu_9_S_5_/TiO_2_ a superior photocatalyst for CO_2_ reduction, especially for selective methane production. The strong adsorption of CO and CHO, coupled with the improved electronic structure, is crucial for its enhanced photocatalytic activity.

## 3. Conclusions

In summary, hydrazine-engineered Cu_9_S_5_/TiO_2_ gel composites were successfully fabricated via a hydrothermal method using gel-derived TiO_2_ as a porous framework. The resulting materials exhibited well-dispersed Cu_9_S_5_ nanocrystals and enhanced crystallinity, which significantly promoted CO_2_ adsorption and photocatalytic activity. The optimized 0.36Cu_9_S_5_/TiO_2_ composite achieved an outstanding methane production rate of 34 μmol·g^−1^·h^−1^ and CO production of 15 μmol·g^−1^·h^−1^, with a methane selectivity of 64.76% and an electron-based CH_4_ selectivity of 88.02%. In contrast, the Cu_9_S_8_/TiO_2_ control sample prepared without hydrazine hydrate generated only CO, indicating the critical role of hydrazine in phase regulation and activity enhancement. The improved performance of Cu_9_S_5_/TiO_2_ is attributed to synergistic effects between the gel-derived TiO_2_ matrix and hydrazine-modulated Cu_9_S_5_ domains. The hydrazine-assisted synthesis not only facilitated Cu_9_S_5_ phase formation and dispersion, but also optimized interfacial electronic structure, thereby enhancing charge carrier separation and intermediate adsorption. DFT calculations confirmed that Cu_9_S_5_/TiO_2_ lowered the energy barriers for key intermediates (*COOH, *CO, and *CHO), enabling efficient methane formation via a deep reduction pathway. This study highlights the potential of gel-based composite photocatalysts for coupling CO_2_ adsorption and selective conversion. The design strategy demonstrated here provides valuable insights into phase and interface engineering for efficient CO_2_-to-CH_4_ photoreduction, contributing to the development of sustainable energy and environmental technologies.

## 4. Materials and Methods

### 4.1. Materials

All reagents used in this study were of analytical grade and used without further purification. Titanium isopropoxide (TTIP), glacial acetic acid, nitric acid (HNO_3_), and isopropyl alcohol (IPA) were purchased from Sinopharm Chemical Reagent Co., Ltd. (Shanghai, China). Sodium hydroxide (NaOH, 0.1022 mol/L) was self-prepared in the laboratory. Thiourea (C_2_H_5_NS, AR, 99%) was purchased from Shanghai Maike Chemical Technology Co., Ltd. (Shanghai, China). Hydrazine hydrate (N_2_H_4_·H_2_O, AR) was obtained from the Pharmaceutical Group Chemistry Reagent Co., Ltd. (Beijing, China). Deionized water (H_2_O) was used for all synthesis, dispersion, and washing procedures. Ethanol (C_2_H_5_OH, AR) was purchased from Guangdong Linmin Chemical Reagent Co., Ltd. (Guangzhou, China). Copper Citrate (C_6_H_5_Cu_2_O_7_, AR) was obtained from Shanghai Maike Chemical Technology Co., Ltd. (Shanghai, China).

### 4.2. Microwave-Assisted Sol-Gel TiO_2_ and CuSx/TiO_2_

#### 4.2.1. Synthesis of Gel-Derived TiO_2_

The synthesis of TiO_2_: The TiO_2_ was prepared using a microwave-assisted sol–gel method to enhance uniformity [31]. Firstly, a homogeneous solution was prepared by mixing 4.8 mL of TTIP, 0.8 mL of glacial acetic acid, and 4 mL of IPA. Then, an acidic solution was prepared by heating 11 mL of Milli-Q water and 0.20 mL of concentrated nitric acid to 90 °C. The TTIP-containing solution was quickly added to the acidified water under vigorous stirring, resulting in a homogeneous milky white reaction mixture. This reaction mixture was transferred to a 60 mL microwave reaction vessel, and water was added to dilute the solution to a final volume of 44 mL. The mixture was irradiated in a microwave reactor (Sineo Microwave Chemistry Technology (ShangHai) Co., ltd. (Shanghai, China), UWave-2000) at 90 °C with a stirring rate of 1200 rpm for 10 min, resulting in a transparent TiO_2_ hydrosol. Finally, the synthesized TiO_2_ hydrosol was freeze dried to obtain loosely packed TiO_2_ aggregates as the final product. The synthesis of TiO_2_ involves the hydrolysis and condensation of titanium alkoxide (such as titanium isopropoxide) in the presence of an acid or base. It may follow the chemical reaction below:

TTIP + H_2_O → TiO_2_ + IPA

In this reaction, TTIP undergoes hydrolysis in the presence of water, forming TiO_2_ and IPA as a byproduct.

#### 4.2.2. Synthesis of Cu_9_S_5_/TiO_2_ Composites

A series of Cu_9_S_5_/TiO_2_ composites with varying Cu loadings (0.12, 0.24, 0.36, and 0.48 mmol) were prepared. For comparison, a control sample without hydrazine hydrate was also synthesized (yielding Cu_9_S_8_/TiO_2_).

Typically, 12 mmol of the synthesized TiO_2_ was dispersed in 50 mL of deionized water and ultrasonicated for 30 min to form a uniform suspension (designated as Suspension A). Meanwhile, aqueous copper citrate solutions of different concentrations were prepared (Solution B) and slowly added dropwise into Suspension A under continuous stirring. The mixture was stirred magnetically for 20 h to ensure sufficient interaction between TiO_2_ and Cu species. Subsequently, 40 mL of 16.8 mM NaOH solution was added dropwise to adjust the pH and facilitate precursor precipitation, followed by 20 min of stirring. Afterward, the precipitates were separated by centrifugation, washed several times with deionized water, and redispersed in 60 mL of 8 mM thiourea solution. For Cu_9_S_5_ formation, 1 mL of 85% hydrazine hydrate was introduced into the dispersion. The resulting mixture was sealed in a 100 mL Teflon-lined reactor and heated at 160 °C for 7 h. Upon cooling, the final solid products were collected by centrifugation, thoroughly washed with anhydrous ethanol and deionized water, and dried in a vacuum oven at 70 °C. The as-obtained products were denoted as 0.12Cu_9_S_5_/TiO_2_, 0.24Cu_9_S_5_/TiO_2_, 0.36Cu_9_S_5_/TiO_2_, and 0.48Cu_9_S_5_/TiO_2_. The control sample synthesized under identical conditions but without hydrazine hydrate was labeled 0.36Cu_9_S_8_/TiO_2_.

In the procedure above, the copper sulfide (Cu_9_S_8_) was formed through the following reactions:

9Cu^2+^ + 8S^2−^ → Cu_9_S_8_

Subsequently, hydrazine hydrate was added as a reducing agent, contributing to a small amount of reduced Cu^+^, and then converting Cu_9_S_8_ into Cu_9_S_5_. The hydrazine reduction process can be represented by the following reactions:

9Cu^2+^ + N_2_H_4_ + 5S^2−^ → Cu_9_S_5_ + N_2_ + 4H^+^

### 4.3. Sample Characterization

The morphology and internal nanostructure of the prepared catalysts were investigated by scanning electron microscopy (SEM; Regulus 8100, Hitachi, (Tokyo, Japan) and high-resolution transmission electron microscopy (HRTEM; Talos F200X, Thermo Fisher Scientific, Waltham, MA, USA). Prior to HRTEM analysis, the samples were dispersed in anhydrous ethanol through ultrasonication, drop-cast onto carbon-supported copper grids, and naturally dried in air. Imaging was conducted at an accelerating voltage of 200 kV. Phase composition and crystallinity were determined via X-ray diffraction (XRD) using a SmartLab diffractometer (Rigaku, Tokyo, Japan) equipped with a Cu Kα radiation source (λ = 1.5406 Å). The measurements were conducted under conditions of 40 kV tube voltage and 30 mA current. Diffraction patterns were collected over a 2θ range of 5–90°, with a step increment of 0.02° and a scan speed of 10° per minute. Photoluminescence (PL) spectroscopy was used to evaluate the recombination characteristics of photogenerated carriers. The spectra were obtained on an FLS980 spectrometer (Edinburgh Instruments, Livingston, UK) under excitation at 325 nm.

Photoelectrochemical performance was assessed using a CHI660D electrochemical workstation (CH Instruments, Shanghai, China) in a conventional three-electrode configuration. For electrode preparation, 5 mg of catalyst was ultrasonically dispersed in a mixture of 2 mL ethanol and 10 μL of 5 wt% Nafion solution. A 100 μL aliquot of the resulting dispersion was deposited onto a 1 cm^2^ indium tin oxide (ITO) glass slide and dried under ambient conditions. The working electrode was coupled with a platinum foil counter electrode and an Ag/AgCl reference electrode. All measurements were carried out in 0.5 M Na_2_SO_4_ electrolyte under light-off conditions unless otherwise specified.

### 4.4. Evaluation of CO_2_ Photocatalytic Reduction

The photocatalytic CO_2_ reduction performance was investigated using a CEL-PAEM-D88P photocatalysis analysis system (Au-light, Beijing China Education Au-light Technology Co., Ltd., (Beijing, China). For each test, 7 mg of photocatalyst was ultrasonically suspended in 25 mL of deionized water to ensure uniform dispersion. The suspension was then vacuum filtered through a Teflon membrane (pore size < 100 μm), and the resulting thin film was dried under vacuum at 60 °C for 30 min.

The prepared catalyst membrane was fixed onto a stainless-steel holder and placed at the center of a gas-tight quartz reaction cell. To provide a humid environment, 5 mL of deionized water was added to the base of the reactor. The chamber was then sealed and purged three times with high-purity CO_2_ (99.999%) to eliminate residual air. After degassing, CO_2_ was introduced to adjust the internal pressure to ~40 kPa. The reaction system was allowed to stabilize for 30 min using a built-in plunger pump to ensure homogeneous mixing of CO_2_ and water vapor. A 300 W xenon arc lamp (PLS-SXE300+, Au-light, China) was employed as the irradiation source to simulate sunlight. The reactor temperature was kept constant at 5 °C throughout the reaction using a circulating water-cooling system to suppress thermal side effects. Evolved gas-phase products were continuously monitored using an integrated online gas chromatograph equipped with one thermal conductivity detector (TCD) and two flame ionization detectors (FIDs). This configuration enabled real-time, simultaneous detection of H_2_, CO, CH_4_, C_2_H_4_, and C_2_H_6_ under ambient pressure.

### 4.5. Calculation of Product Yield and Selectivity

The generation rates of gaseous products (H_2_, CO, CH_4_, C_2_H_4_, and C_2_H_6_) were quantified based on their concentrations measured by gas chromatography and normalized to catalyst mass and irradiation time. The formation rate (R) of a given product was calculated using the following equation:

(1) $$R=\frac{n}{m\times t}$$

where R is the product yield (μmol g^−1^ h^−1^), n is the amount of product formed (μmol), m is the mass of photocatalyst used (g), and t is the reaction time (h).

To evaluate the distribution of reduction products, the product selectivity (S) toward species was determined by

(2) $$S_{x}=\frac{n_{x}}{\sum n_{i}}\times 100\%$$

where $n_{x}$ is the amount of product, and $n_{i}$ is the total amount of all carbon-containing reduction products (e.g., CO, CH_4_, C_2_H_4_, C_2_H_6_).

For further insight into the reduction pathway, the electron selectivity was calculated based on the number of electrons transferred per molecule of each product. The electron-based selectivity toward CH_4_, for instance, is expressed as

(3) $$e^{-}\text{-}{Selectivity}_{{CH}_{4}}=\frac{8\;\times \;n_{{CH}_{4}}}{\sum \left(e_{i}\;\times \;n_{i}\right)}\;\times \;100\%$$

where $e_{i}$ is the number of electrons required for the formation of product i (e.g., 2 for CO, 8 for CH_4_, 12 for C_2_H_4_, etc.), and $n_{i}$ is the molar quantity of each product.

All values were derived from the integrated GC peak areas using calibrated response factors.

### 4.6. DFT Calculation Method

First-principles calculations based on density functional theory (DFT) were performed using the Vienna Ab initio Simulation Package (VASP, version 5.4.4). The interactions between core and valence electrons were described using the projector-augmented wave (PAW) method [35,36,37]. A plane-wave cutoff energy of 400 eV was applied for all calculations. The electronic self-consistent field (SCF) iterations were considered converged when the total energy change was below 1 × 10^−5^ eV. Geometry optimizations were carried out until the residual atomic forces were reduced below 0.03 eV·Å^−1^. To investigate the interfacial behavior of adsorbed intermediates involved in CO_2_ photoreduction, surface models were constructed for the TiO_2_ (101) and CuSx/TiO_2_ heterojunction interfaces. Each model was built using a four-layer slab structure, where the two bottom layers were fixed during both structural optimization and transition state calculations. A vacuum space of 15 Å was introduced perpendicular to the slab to eliminate interlayer interactions between periodic images. Thermodynamic properties and reaction energetics were obtained by computing the Gibbs free energy (G), which includes electronic, vibrational, enthalpic, and entropic contributions. The Gibbs free energy of a given species A was estimated using the following expression:

(4) $$G_{A(T,\;p)}\;=\;E_{\mathrm{total},\;A}\;+\;E_{\mathrm{ZPE}}\;+\;∆H(0\;\to \;T)-TS(T,\;P)$$

in which *E*_total, A_, *E*_ZPE_, ∆*H*(0 → *T*), and *S*(*T*, *P*) are the total energy obtained by DFT calculation, the zero-point energy, the enthalpy change from 0 K to temperature T, and the entropy at temperature T and pressure P, respectively. All thermodynamic parameters were evaluated using the VASPKIT 1.3.6 post-processing toolkit [38].

## Data Availability

The data presented in this study are openly available in article.
